# Immune-Related Gene Expression and Cytokine Secretion Is Reduced Among African American Colon Cancer Patients

**DOI:** 10.3389/fonc.2020.01498

**Published:** 2020-09-02

**Authors:** Jenny Paredes, Jovanny Zabaleta, Jone Garai, Ping Ji, Sayed Imtiaz, Marzia Spagnardi, Joussette Alvarado, Li Li, Mubarak Akadri, Kaylene Barrera, Maria Munoz-Sagastibelza, Raavi Gupta, Mohamed Alshal, Maksim Agaronov, Henry Talus, Xuefeng Wang, John M. Carethers, Jennie L. Williams, Laura A. Martello

**Affiliations:** ^1^Division of Gastroenterology and Hepatology, Department of Medicine, SUNY Downstate Medical Center, Brooklyn, NY, United States; ^2^Department of Pediatrics, Louisiana State University Health Sciences Center, New Orleans, LA, United States; ^3^Stanley S. Scott Cancer Center, Louisiana State University Health Sciences Center, New Orleans, LA, United States; ^4^Department of Family, Population and Preventive Medicine, Stony Brook University, Stony Brook, NY, United States; ^5^Department of Surgery, SUNY Downstate Medical Center, Brooklyn, NY, United States; ^6^Department of Genetics, Louisiana State University Health Sciences Center, Loyola University New Orleans, New Orleans, LA, United States; ^7^Department of Pathology, SUNY Downstate Medical Center, Brooklyn, NY, United States; ^8^Department of Pathology, Kings County Hospital, Brooklyn, NY, United States; ^9^Department of Surgery, Kings County Hospital, Brooklyn, NY, United States; ^10^Department of Biostatistics and Bioinformatics, H. Lee Moffitt Cancer Center & Research Institute, Tampa, FL, United States; ^11^Department of Internal Medicine, University of Michigan, Ann Arbor, MI, United States

**Keywords:** African American, cancer disparities, colon cancer, cytokines, RNASeq, Nanostring

## Abstract

**Background:** Colorectal cancer is the third most deadly cancer among African Americans (AA). When compared to Caucasian Americans (CA), AA present with more advanced disease and lower survival rates. Here, we investigated if differences in tumor immunology could be contributive to disparities observed between these populations.

**Methods:** We examined gene expression of tumor and non-tumor adjacent tissues from AA and CA by whole transcriptome sequencing, and generated scores for immune cell populations by NanoString. In addition, we utilized “The Cancer Genome Atlas” (TCGA) database from AA and CA as a validation cohort. Finally, we measured the secretion of cytokines characteristic of effector T helper cell (T_h_) subsets by ELISA using plasma from each AA and CA participant.

**Results:** Colon tumors from AA patients showed significant fold-change increase in gene expression when compared to CA for *FOXP3* (6.22 vs. 3.22), *IL1B* (103 vs. 11.4) and *IL8* (220 vs. 28.9) (*p* < 0.05). In contrast, among CA we observed statistically higher gene expression of markers associated with antitumor activity such as *GZMB* (Granzyme B), *IFNG* and the immunotherapy targets *PDL1* (*CD274*) and *CTLA4* (*p* < 0.05). TCGA data validated our observed higher gene expression of *GZMB* and *PDL1* in CA patients when compared to AA. Notably, our observations on immune cell populations show that AA tumors have significantly higher number of exhausted CD8+ cells (*p* < 0.01), mast cells (*p* < 0.02) and increased T regulatory cells when compared to CA. AA colon cancer patients differed from CA in cytokine production patterns in plasma (i.e., reduced IL-12).

**Conclusions:** Our study demonstrates significant differences of the immunological profiles of colon tumors from AA compared to CA that suggest a deficiency of appropriate immune defense mechanisms in terms of gene expression, recruitment of immune cells and systemic secretion of cytokines. As such, these immune differences could be mitigated through population-specific therapeutic approaches.

## Introduction

Colorectal cancer (CRC) is the third most deadly cancer in the U.S., with higher mortality rates for African Americans (AA) than the rest of the general population (1, 2). Publications on genome-wide association studies of AAs vs. Caucasian Americans (CA), Hispanics, and Asians have concluded that there are some risk variants for colon cancer unique to African Americans (3). Additionally, previous findings suggest genetic differences between tumors from AA when compared with CA (4), including higher frequency of *KRAS* mutations and unique somatic alterations in the *BRAF, KRAS*, and *PIK3CA* genes, and potential somatic driver genes such as *EPHA6* and *FLCN* (4–6). Understanding unique genetic alterations in colorectal tumors from AAs may allow personalized medicine approaches that could lead to better outcomes in this population.

Furthermore, Wang et al. reported that tumor DNA from AA CRC patients presented a significant difference in the number of methylated regions when compared with CRC tumors from CA patients (7), including four anti-inflammatory genes (*NELL1, GDF1, ARHGEF4*, and *ITGA4*) that could lead to differences in the inflammatory state of tumors from AA CRC patients (7). In addition, Sanabria-Salas et al. recently found a significant association between a haplotype on the Interleukin 1B gene, African ancestry, and CRC risk (8), underscoring the potential role of genetic ancestry and cytokine profile in colon cancer progression. These findings align with results by Basa et al. which demonstrated that CRCs tissues from AAs have lower infiltration of activated T and Natural Killer (NK) cells that express cytotoxic granzyme B (GzmB) than CRCs from CAs (9). Although these studies pinpoint differences in the tumors of AA and CA CRC patients, genetic factors that may contribute to inflammation and evasion of immune checkpoints and cellular antitumor activity in colon tumors from AAs have yet to be explored collectively.

Taking into consideration the potential contribution of cytokines in the development of colon cancer (10), we aimed to investigate the correlation of the expression of cytokine genes and secretion with the population of immune cells in colon tumors from AA patients. Cytokines that promote colon tumor development include the tumor necrosis factor (TNF), interleukin 6 (IL-6), interleukin 1B (IL-1B), and interleukin 8 (IL-8). In contrast, cytokines such as interleukin 10 (IL-10) and transforming growth factor B (TGF-B) inhibit colorectal tumorigenesis. Additional ways in which cytokines promote colon cancer is by modulating the recruitment and activation of immune cells at the tumor site. Pro-inflammatory cytokines are produced by macrophages and dendritic cells (DC) during early states of cancer development or by T cells during late-stage tumor progression (10), examples such as T helper 1 (Th1) and T helper 17 (Th17) cells (11).

In addition, since immune checkpoint inhibitors were the first immune cell mediated therapies to be implemented for the treatment of colon cancer (12–14), we wanted to elucidate the gene expression of immunotherapies' targets in colon tumors from AA patients. Tumor cells counteract and avoid immune detection and destruction, by expressing cell surface inhibitory checkpoint molecules. For example, programmed death-1 (PD-1) and cytotoxic T lymphocyte associated antigen 4 (CTLA-4). Hence, blocking the PD-1/PD-L1 and CD80/CTLA-4 checkpoints interactions has proven to be an effective immunotherapy approach (13, 14). Lastly, as colon cancer tumors are also infiltrated by neutrophils, mast cells, and myeloid-derived cells, among others; we observed the recruitment of cell populations that may also suppress antitumor immune responses at the tumor site.

In this study, we utilized gene expression profiles (RNA-seq) and NanoString technology to demonstrate that CRC tumors from AA patients differ from CA patients in their gene expression patterns and immune cell recruitment, particularly those associated with T regulatory cells (higher *FOXP3*), pro-inflammatory cytokines (higher *IL8* and *IL1B*), markers for cellular antitumor activity (lower *GZMB* and *IFNG*), as well as targets for immunotherapies (lower *PDL1* and *CTLA4*). Our gene expression results correlated with significantly higher numbers of a subset of CD8+ T cells (exhausted), T regulatory cells and myeloid cells in the AA cohort as evidenced by NanoString. The levels and patterns of cytokines associated with T cell activation and differentiation secreted into the plasma of AA colon cancer patients significantly diverged from those observed for CA patients. Genomic data from The Cancer Genome Atlas (TCGA) showed agreement and validated our observation that AA patients have significantly lower expression levels of *PDL1* and *GZMB* compared with CA patients.

## Materials and Methods

### Human Samples

This study was approved by the SUNY Downstate and Kings County Institutional Review Boards (Protocol #312509). Male and female patients with a suspected diagnosis of colorectal adenocarcinoma from SUNY Downstate Medical Center and Kings County Hospital aged 18 years and older that self-identified as African American or Afro-Caribbean (AA) were consented for participation in our study. Patients who self-identified as Caucasian American (CA), meeting the same criteria, were consented from Stony Brook University. For the purpose of evaluating the immunological profile of tumors and plasma of these patients without potential confounders, we excluded patients with known infectious diseases such as HIV and hepatitis, patients currently treated with immunosuppressive drugs or antibiotics, and patients diagnosed with Crohn's disease or ulcerative colitis. In order to study naive tumors, only patients in which colon cancer was their primary cancer at the time of diagnosis were included and patients treated with neoadjuvant chemotherapies were excluded. In addition, since treatment for rectal cancer differs from colon cancer (i.e., regimen of radiation) we excluded rectal cancers from our research design. Lastly, as we aimed to study sporadic colon cancer, we excluded patients diagnosed with Lynch syndrome or patients with familial adenomatous polyposis disease.

Colon tissues were collected in DMEM media (Gibco, Carlsbad, CA) with penicillin-streptomycin (Thermo Fisher Scientific, Fair Lawn, NJ). Tumor and matching adjacent non-tumor tissues were transferred to RNAlater (Qiagen, Germantown, MD) and kept at −80°C for future analysis. Ten milliliter of blood was collected from each participant on the day of the surgery in blood collection tubes (vacutainer tube with EDTA) from BD (San Diego, CA). Blood was spun (1,100 g × 10 min, 25°C) to separate plasma from the buffy coat and red blood cells. Plasma samples were kept at −80°C for further analysis.

### RNA Sequencing

RNA sequencing and analysis was done at the Translational Genomics Core (TGC) at the Stanley S. Scott Cancer Center, LSUHSC, New Orleans, LA. RNA was isolated from tumor and adjacent non-tumor (control) tissues using the Universal RNA/DNA Isolation kit (Qiagen, Germantown, MD) following the manufacturer's protocol. Isolated RNA was assessed using a Qubit (ThermoFisher, Waltham, MA) and checked for RNA integrity on the Agilent Bio Analyzer 2100 (Agilent, Santa Clara, CA). Paired-end libraries (2 × 75) were prepared (600 ng per sample) using the TruSeq Stranded mRNA Library Prep kit, validated, and normalized following the recommendations of the manufacturer (Illumina, San Diego, CA). Libraries were sequenced in the NextSeq500 using a High Output Kit v2.5, 150 cycles from Illumina. FASTQ output files were uploaded to Partek Flow, contaminants (rDNA, tRNA, mtrDNA) were removed using Bowtie2 (version 2.2.5) and the unaligned reads were then aligned to STAR (version 2.5.3a) using the hg19 version of the human genome as reference. Aligned reads were quantified to the hg19-Ensembl Transcript release 75 and normalized by log2 (x+1) transformation. Normalized counts were used to determine differential gene expression between tumor and non-tumor (control) with gene specific analysis (GSA). All analyses were corrected for multiple comparisons at a false discovery rate (FDR) of 0.05 (at least). Heatmaps were generated using the embedded algorithm in Partek Flow for hierarchical unsupervised comparison of the samples using Euclidian distance. Gene Ontology (GO) enrichment and pathway analysis (with embedded KEGG function) also were done in Partek Flow. Raw data (FASTQ) has been uploaded to the Gene Expression Omnibus (GEO) with the accession number GSE146009.

### NanoString

RNA from tumor tissues (same source as RNAseq) from AA and CA individuals with colon cancer were used for the unbiased detection of 770 immune-related genes using the CancerImmune panel (NanoString). RNA (50 ng at 10 ng/μl) was mixed with Capture and Reporter probes (NanoString) and incubated for 18 h at 65°C after which the sample was run in a nCounter cartridge (NanoString). Detection was done in the SPRINT nCounter system (NanoString) at the TGC. Cell population estimation was done by using the “cell type profiling” algorithm in the nSolver software v4.0 (NanoString). Estimation of cell population is based on the expression of gene patterns specific to each population after normalizing to seven housekeeping genes.

### Real-Time PCR

To validate the immune cell infiltration, we used real-time PCR to determine the expression of the immune cell marker genes. We used primer-probes sets (TaqMan) from Thermo Scientific to detect *S100A2* (PMN), *GZMB* (exhausted CD8 cells), *FOXP3* (T-regs) and *IL1B* as a marker of Th1 inflammatory responses. We used the 2^−ΔΔCT^ method to determine the relative expression of those genes in tumor tissues using *GAPDH* as the housekeeping gene.

### Enzyme-Linked Immunosorbent Assays

Cytokines associated with T_h_ subsets (T_h_1, T_h_2, and T_h_17) and inflammation were measured in the plasma from 20 AA and 20 CA colon cancer patients using Multi-Analyte kits from Qiagen (Germantown, MD): T_h_1 (IL-12, IL-5, TNF-α, IL-2, INF-γ), T_h_2 (IL-13, IL-4, IL-5, IL-3, IL-10) and T_h_17 (IL-17A, IL-1A/B, IL-9). Statistical analysis was done by *T*-test, *P* < 0.05 for significance using Microsoft Office, Excel, 2016 (Redmond, WA).

### TCGA Colon Adenocarcinoma Data Analysis

From the TCGA colon adenocarcinoma (COAD) gene expression data, we downloaded processed RNAseq data (level 3 normalized RNAseq by Expectation-Maximization from the Broad Institute Genome Data Analysis Center Firehose portal (http://firebrowse.org) (data version 2016_01_28). We removed all non-tumor samples and applied the log2(x+1) transformation to RSEM values for all analyses and plots. The TCGA clinical file was downloaded from UCSC Xena data hubs (version 11-27-2017) and was matched with the gene expression data using the unique TCGA barcodes. The comparison of gene expression data between the two racial/ethnic groups was made using the Wilcoxon (*non-parametric*) test. Patients in each ethnic group were categorized into high and low gene (*GZMB*) expression subgroups using the median gene expression value for each ethnic group. Overall survival (OS) of patients in gene expression subgroups was compared using Kaplan169 Meier plots and log-rank tests.

## Results

### Colon Cancers From African and Caucasian Americans Differ in the Expression of Immune-Related Genes

**Figure 1:**
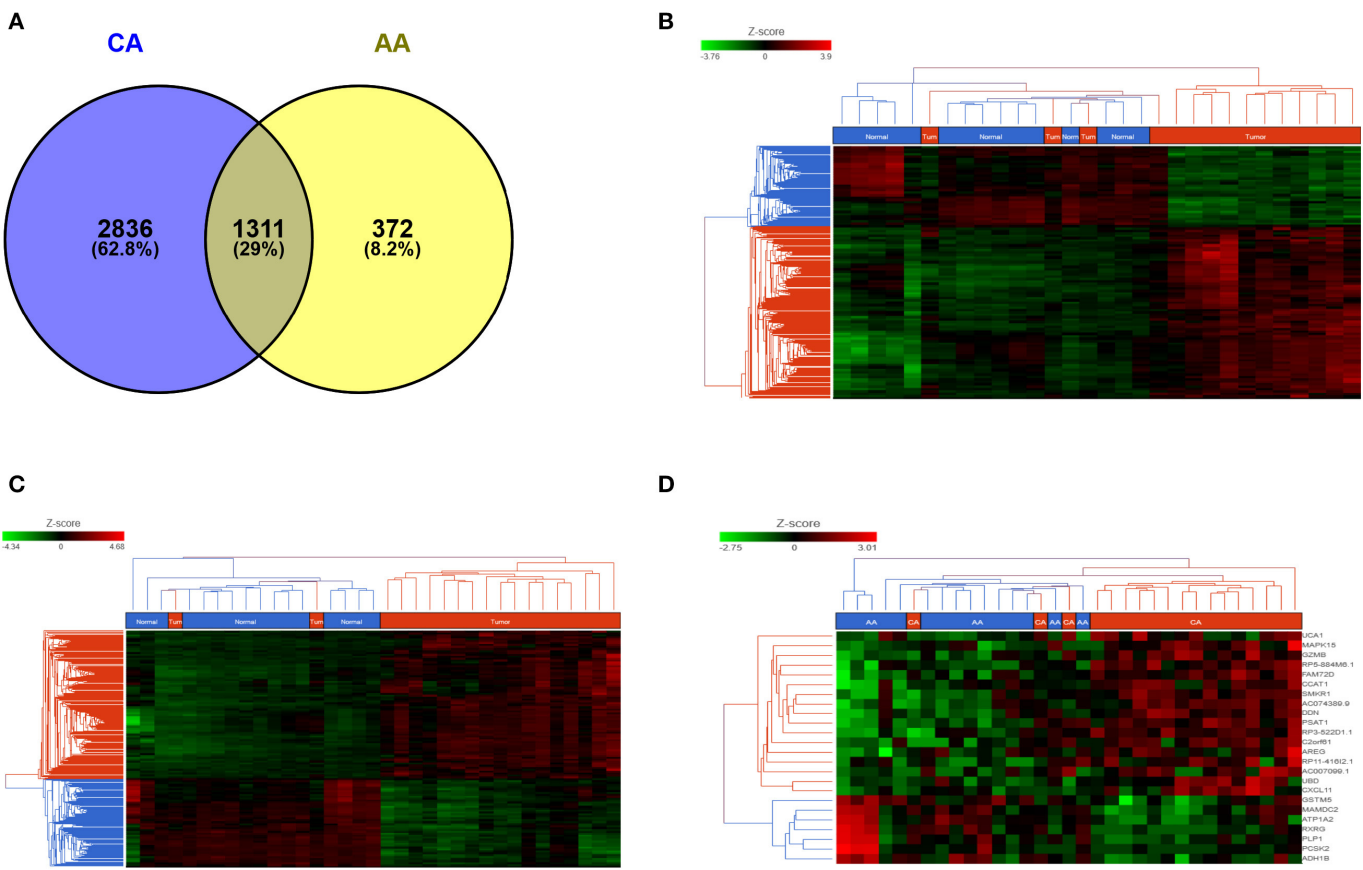
AA and CA tumors present different gene expression patterns. **(A)** Venn diagram depicting the number of common and unique genes in AA and CA colon tumors (compared to their respective non-tumor tissues). Analysis adjusted for multiple comparisons using FDR < 0.05 and fold change >2; Unsupervised hierarchical clustering of genes in AA **(B)** and CA **(C)** tumor tissues. Heat map for AA was generated using FDR < 0.05 and fold change >2; heat map for CA was generated at FDR < 0.05 and fold change >2; **(D)** Common genes (*n* = 1,311) between Tumor vs. non-tumor tissues (FDR < 0.05, fold change >3) in AA and CA were used to identify genes that may differentiate both tumor tissues from both ethnicities.

We studied the whole transcriptome profile of colon cancer tissues from African American (15 tumors and 15 adjacent non-tumor tissues, *n* = 30) and Caucasian American (18 tumors and 17 adjacent non-tumor tissues (*n* = 35). Information regarding race/ethnicity, age, and tumor stage of the patients is summarized in Supplementary Table 1. Differential gene expression analyses (*FDR* < 0.05) indicate that, compared to their own non-tumor counterpart, the gene profiles of tumors from AA patients uniquely expressed 372 genes (8.2%) whereas CA tumors significantly expressed 2,836 genes (62.8%) (Figure 1A). Unsupervised hierarchical clustering of samples from AA (*FDR* < 0.05, fold change 2) and CA (*FDR* < 0.05, fold change 2) clearly illustrate that, the gene expression patterns are distinct between tumor (red bar) and non-tumor tissues (control, blue bar) for both cohorts (Figures 1B,C, respectively).

In order to objectively evaluate those genes that were commonly and significantly expressed in the tumors of both AA and CA patients, we focused on the common genes originally identified at FDR < 0.05 and fold change >2. To determine the differential expression of these common genes, if any, between AA and CA tumor tissues, we used Partek Flow analysis. We identified 218 genes differentially expressed at FDR < 0.05. Of these genes 141 were expressed at *p* < 0.05, 68 at *p* < 0.001 and 9 at *p* < 0.0001. The heatmap Figure 1D (FDR < 0.05 and fold change >3) indicate that those genes, including *PSAT1* (*p* = 5.1 × 10^−6^), *RP5-884M6.1* (*p* = 1.3 × 10^−5^), *FAM72D* (*p* = 3.3 × 10^−5^), *GZMB* (*p* = 6.4 × 10^−5^), and can differentiate tumors from AA and CA patients.

We found that the immune-related gene encoding granzyme B (*GZMB*), a protease secreted by cytotoxic T and natural killer (NK) cells with anti-tumoral properties (15) was among the most differentially expressed gene between the two cohorts. *GZMB* was up-regulated in CA tumors and down-regulated in AA tumors when compared to their matching non-tumor controls. Another differentially down-regulated gene in AA tumors was *NEIL3* (not shown in the heatmap), which encodes the Neil Like DNA Glycosylase 3 that repairs oxidative lesions in telomeres due to oxidative stress and belongs to the base excision repair pathway (15). Remarkably, AA tumors show a distinct pattern in gene expression in terms of immunological differences between tumor and adjacent non-tumor tissues when compared to CA tumors. These variations, including those in *GZMB, CXCL11*, and *NEIL3* suggest that the differences between patients from both races could be related to their immune response at the tumor niche.

We examined the expression levels of genes that may play a role in the activation of Granzyme B secreting Cytotoxic T cells. As shown in Table 1, AA tumors had more than 4-fold lower levels of *GZMB* when compared to CAs. Interestingly, we did not find expression of the main *GZMB* inhibitor, *SERPINB9* (15) in AA while it was 2.64-fold-increased in tumor tissues of CA, when compared to non-tumor tissues. When we evaluated the expression of *FOXP3*, a biomarker for T regulatory cells that have the capability of inhibiting cytotoxic T cells (15), we found that *FOXP3* gene expression was close to 2-fold higher in AA tumors compared to CAs. We used real-time PCR to validate the expression of *GZMB*, along with *FOXP3*, and found similar results as in our gene expression assays (Supplementary Figures 2A,B, respectively). *GZMB* gene expression was higher in the CA cohort (*p* = 0.007) and *FOXP3* was significantly upregulated in the tumors from AA patients (*p* = 0.0056). Collectively, the data suggests that the expression level of *GZMB* observed in AAs may not be due to an upstream cell regulator, but instead, may have to do with gene expression patterns of the immune cells at the tumor site.

**Table 1:** Gene expression of *GZMB*, its inhibitor (*SERPINB9*) and the T regulatory cells marker, FOXP3^*^.

**Race**	**Gene**	**Fold change tumor vs. non-tumor**	***P-value***	**FDR**
AA	*GZMB*	2.36	0.003	0.05
CA	*GZMB*	9.67	0.0000006	0.00005
AA	*SERPINB9*	Not found	–	–
CA	*SERPINB9*	2.64	0.0005	0.005
AA	*FOXP3*	6.18	0.0000001	0.0002
CA	*FOXP3*	3.22	0.0005	0.005

* *GZMB, Granzyme B; SERPINB9, Serpin Family B Member 9; FOXP3, Forkhead Box P3. Tumor vs. non-tumor per cohort, p > 0.005, Illumina*.

### Immune Pathway Analysis of Colon Cancers

**Figure 2:**
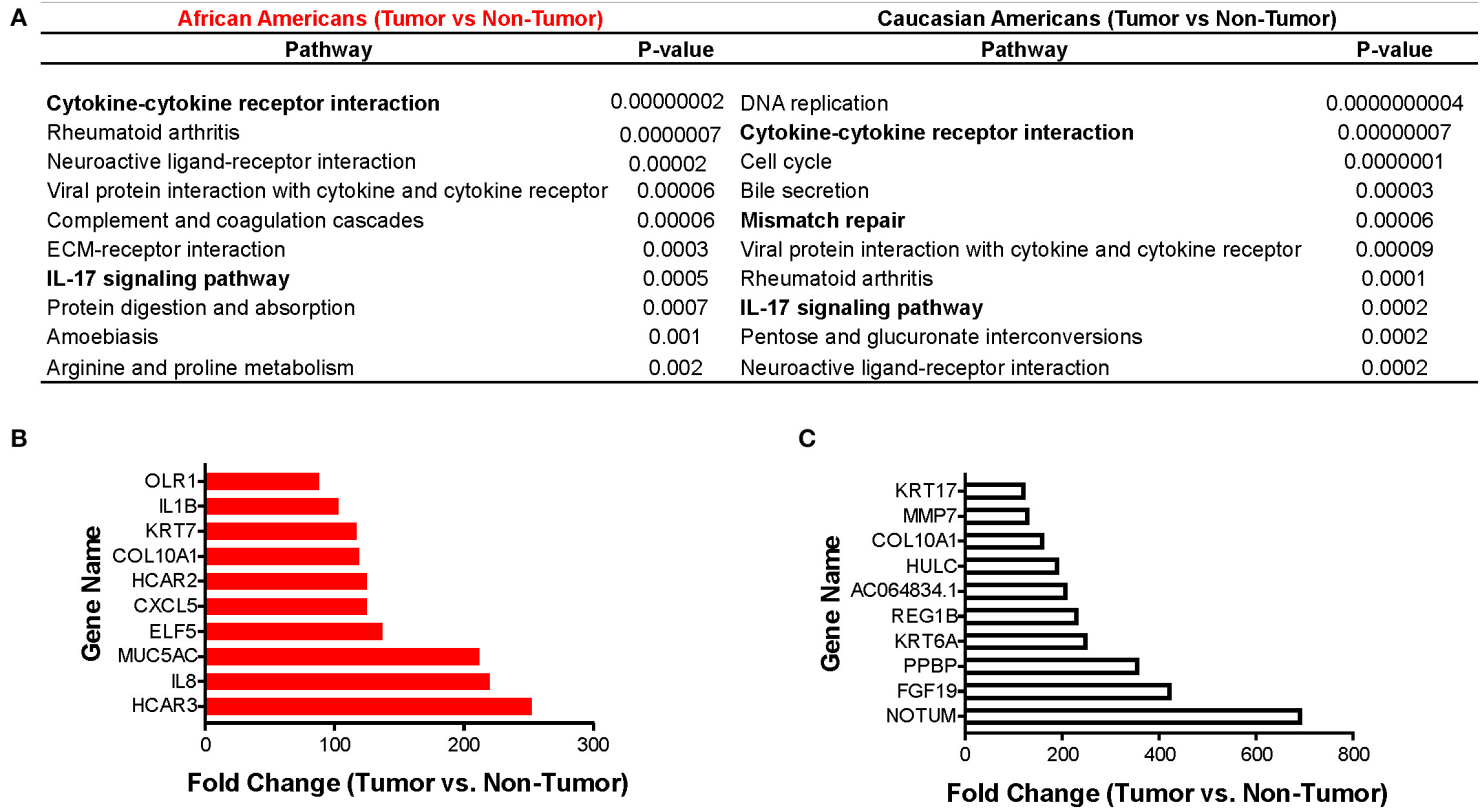
Immune-related pathways in AAs with colon cancer. Using the Pathways Enrichment algorithm in Partek Flow (KEGG) we identified the most significant pathways in which the differentially expressed genes (for both AA and CA) are included. Top 10 genes molecular pathways associated with differentially expressed genes (tumor vs. non-tumor, FDR < 0.05, fold change >2) in African and Caucasian Americans **(A)** with colon cancer (*n* = 65). Genes significantly expressed in tumors from AAs **(B)** and CAs **(C)**, respectively, related to the immune system (fold change over control). *p* < 0.05 for significance.

Using the KEGG algorithm embedded in Partek Flow, we identified the molecular pathways in which those differentially expressed genes between tumor and non-tumor tissues of AA and CA individuals are represented. We found the cytokine-cytokine receptor interaction pathway (Figure 2A and Supplementary Figure 1) as the most significantly expressed in AA tumors when compared to non-tumor controls (*p* = 1.9 × 10^−8^). Gene expression levels from CA tumors (Figure 2B) in turn, had the most significant *p*-value for the DNA replication pathway (*p* = 4 × 10^−10^).

We next investigated if there was a correlation between identified pathways with expression of selective genes (genes that belong to the pathways) between tumor and non-tumor tissues within the cohorts. Figures 2C,D show the 10 genes with the highest fold-change values of expression between tumor and non-tumor tissues for AAs and CAs, respectively. In accordance with our pathway analysis, the greatest differences in terms of fold-change of gene expression for AA include the cytokine genes *IL1B* and *IL8* as well as the chemokine *CXCL5*. The fold-change values for the CA cohort showed *NOTUM, FGF19*, and *PPBP* (as the most significantly changed). *PPBP* encodes for pro-platelet basic protein) which correlates with the DNA replication pathway results from Figure 2A. Collectively, our gene expression profiling analysis suggests that AA and CA colon cancer patients differ in their gene expression in terms of cancer-associated pathways and fold-change values of gene expression between tumor and adjacent non-tumor tissue.

**Figure 3:**
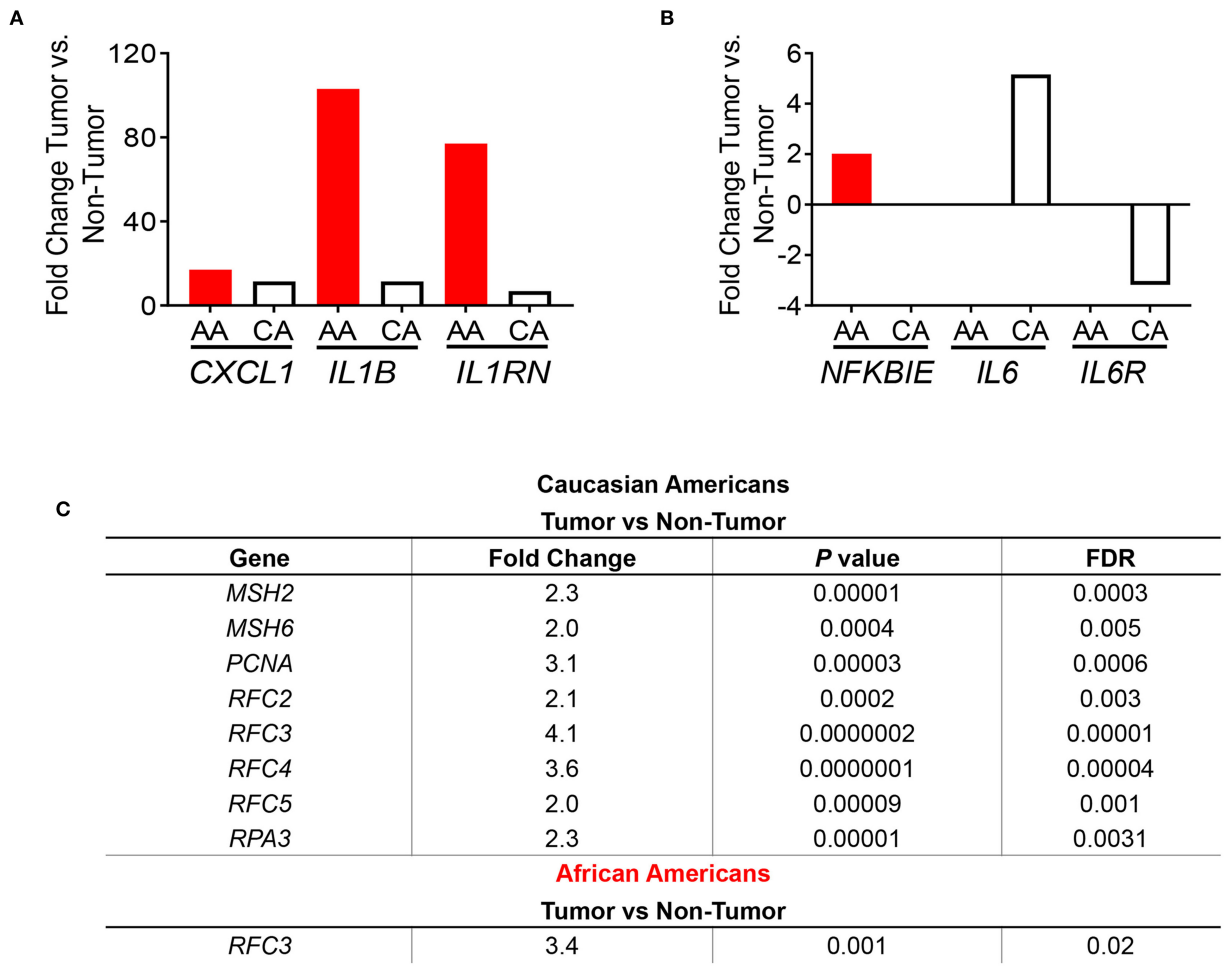
Immune-related gene expression in colon tumors from AA and CA colon cancer patients based on pathways enrichment analysis. **(A)** Comparison of the expression levels of genes from the IL1B pathway between AA and CA patients. **(B)** Expression of genes of the NFKβ pathway. **(C)** CAs present a significant upregulation of the DNA repair pathway and genes involved. Fold change, *p* < 0.05.

After identifying the cytokine-cytokine receptor pathway as the most significantly expressed among AA colon cancers, we assessed the gene expression of cytokines belonging to this immunogenic pathway. In concordance with previous findings from Sanabria-Salas et al. on haplotypes of the *IL1B* gene and CRC risk in populations with African ancestry (8), we observed a 9-fold increase expression of *IL1B*, a 7.6-fold increase in *IL8* expression, and a 3.3-fold increase in *IL1A* in AA patients when compared to CA patients (Table 2). In contrast, we observed that CA tumors expressed 7.3-fold greater upregulation of interferon gamma (*IFNG*), suggestive of cytotoxic T cell activity (16, 17) as compared to AA tumors. Interestingly, several lines of research show a dual pro-and anti-inflammatory effect of IFN-γ in colon cancer (18). Up-regulation of *IL11* in CA tumors when compared to AA tumors was also observed (14.8-fold-increase as compared to AA). Due to the more than 100-fold increase of *IL1B* and *IL8* expression in tumors from AA (relative to their non-tumor counterparts), we investigated if our cohorts differed in *IL1B* gene expression as well as the chemokine genes from the IL-1β pathway, *CXCL1* and *IL1RN*. We hypothesized that increased level of expression in these genes lead to increased IL-1β production. As seen in Figure 3A, these genes had higher expression in tumors from AAs than in CAs, including *IL1RN*, the negative regulator of the receptor for IL-1β and indicative of the activation of its negative feedback loop.

**Table 2:** Cytokines detected in AA tumors^*^.

**Cytokine-cytokine receptor interaction pathway**		**Fold change (Tumor vs. Non-tumor)**	
		**AA**	**CA**
		*p* = 0.0000001	*p* = 0.0000007
Inflammatory	*IL8*	220	28.9
	*IL1B*	103	11.4
	*IL1A*	60.5	18.5
	*IL11*	3	44.4
Anti-Inflammatory	*IFNG*	NS	7.3

* *IL8, Interleukin 8; IL1B, Interleukin 1Beta; IL1A, Interleukin 1Alfa; IL11, Interleukin 11; IFNG, Interferon Gamma. Pathways were generated with KEGG software and gene expression values with RNAseq (Illumina)*.

Subsequently, we examined the expression levels of genes belonging to the NF-kβ pathway, which is downstream of IL-1β and promotes *IL8* gene expression (17). Only the gene *NFKBIE*, which encodes for the *NFKB* inhibitor epsilon protein (17) was significantly differentially expressed in tumors from AAs and CAs. Surprisingly, *NFKBIE* expression was significantly higher only in the AA tumors as no significance was detected in tumors from CAs (Figure 3B) suggesting that the increased expression of the *IL1B* and *IL8* genes are not due to a downregulation of the inhibitor of NF-κB. Furthermore, since NF-κB also promotes expression of IL-6 and its receptor IL6R, we investigated the expression of both genes in each cohort. We observed a non-significant difference in *IL6* gene expression between tumor and non-tumor in AA and a 5.2-fold increase of the cytokine in tumors from CA as well as a non-significant reduction (−3.18 fold) of the IL6R gene only in CA. Collectively, these results suggest that upregulation of the genes *IL1B* and *IL8* may be independent of *NFKBIE* and the *IL6* gene. Lastly, we validated the gene expression of *IL1B* by qPCR. In agreement with our gene expression results, *IL1B* was significantly higher in AA patients (*p* = 0.0374) (Supplementary Figure 2C). These results validate the *IL1B* whole transcriptome findings for the two racial groups investigated.

One of the most significantly expressed genes among tumors from CAs was the DNA Mismatch Repair (MMR) pathway (*p* = 6.14 × 10^−5^) (Figure 2B). This pathway, which was not significantly expressed in tumors from AAs, is linked to the clinical response to currently available immunotherapies (12–14). For that reason, we investigated the gene expression of the MMR genes *MSH2, MSH5, MSH6, PCNA* (Supplementary Figure 3) and other genes that play a role in DNA repair and replication such as *RFC2, RFC3, RFC4, RFC5*, and *RPA3* (Figure 3C). All genes, except *MSH5*, were significantly expressed in CA tumors as compared to control tissues (*p* < 0.05) and at least 2.0-fold over their basal levels. On the contrary, only one of these genes, *RFC3* (replication factor subunit 3), was significantly expressed in colon tumors from AAs. Hence, we aimed to investigate if the observed variables between the cohorts in terms of DNA repair and DNA replication would correlate with differences in microsatellite instability and, with the expression of the *PDL1* and *CTLA4* genes that are associated with MSI status and response to immunotherapies (12).

**Figure 4:**
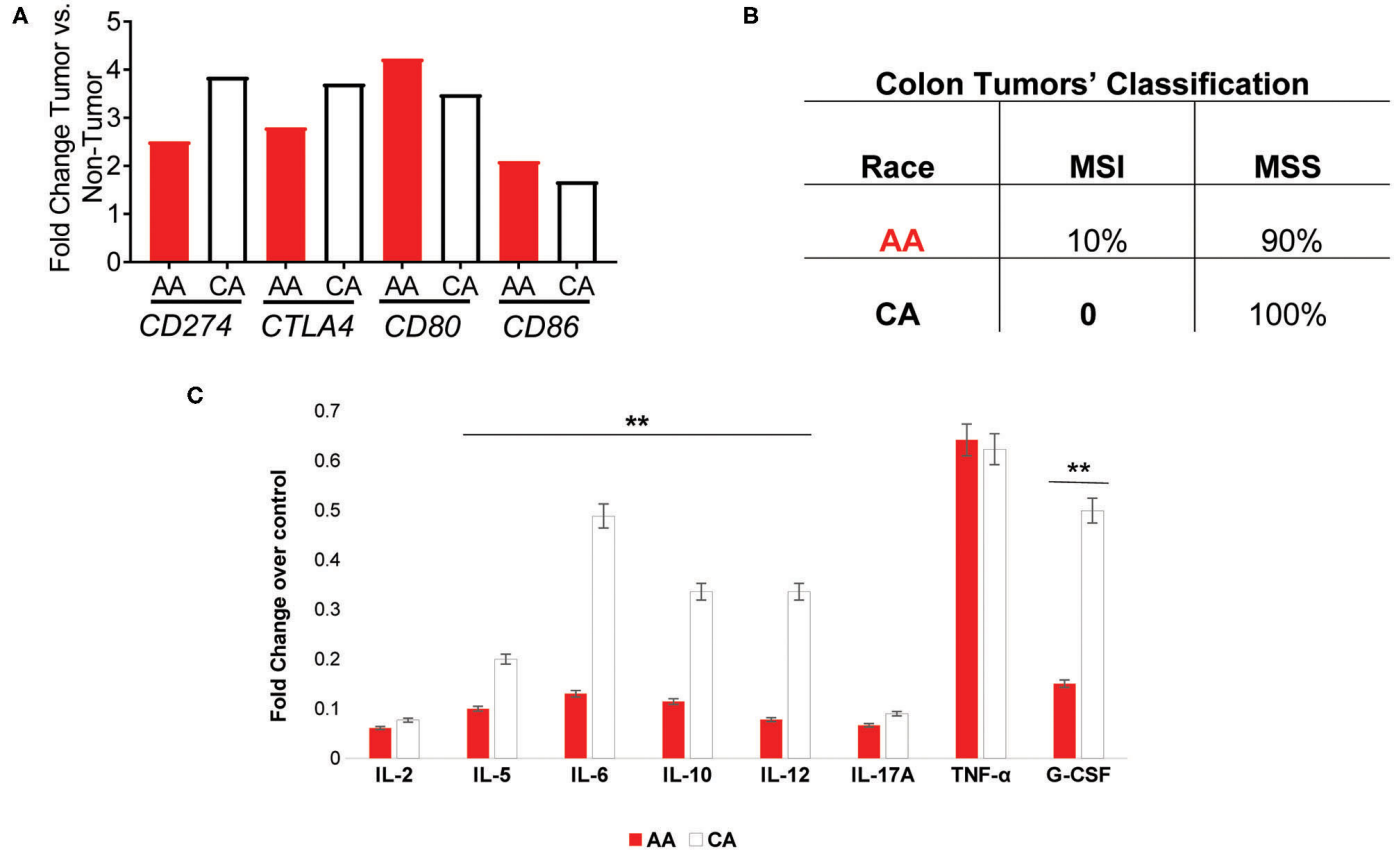
AA and CA tumors differ in their expression in genes associated with immunotherapies, prognostic targets, tumor types, and systemic cytokine expression. **(A)** Genes significantly different between tumor and adjacent non-tumor (control) for the *PDL1* (*CD274*) and *CTLA4* immuno-checkpoint therapies as well as their co-stimulatory molecules, CD80, and CD86. Fold change, *p* < 0.05 **(B)** AA and CA patients differ in the number and type of colon tumors based on genetic stability. **(C)** Systemic cytokine secretion in plasma from AA and CA patients. Significantly, expressed cytokines over the control values provided by the Multi-Analyte, ELISA kit (Qiagen) were selected (*n* = 40). Points of significance between the two cohorts denominated as **p < 0.005.

We examined the expression of the genes that encode for the targets of the currently available immunotherapies for colon cancer, *CD274* (*PDL1*) and *CTLA4* (12–14). We found that both AA and CA tumors have a significantly higher expression, when compared to their non-tumor counterparts, of *CD274* (FDR for AA 0.03, FDR for CA 0.02) but the expression in CA was 35% higher, suggesting that CA patients may have higher likelihood of qualifying for these immunotherapies than AA patients (Figure 4A). Even though the expression of *CTLA4* did not reach significant difference (at the FDR level) between tumor and non-tumor tissues in both AA and CA, its expression was 25% higher in Whites. Lastly, when we investigated the gene expression of the co-stimulatory molecules of these targets, *CD80* and *CD86* (12), we saw higher levels of expression of both genes in the AA cohort with lower levels in the CA group for both targets. It is important to note that only the expression of *CD80* reached significance when comparing tumor vs. no-tumor tissues in both ethnicities. Subsequent to our findings regarding the immunotherapy targets, we assessed the molecular classification of the tumors in our samples as microsatellite instable (MSI) or microsatellite stable (MSS). Figure 4B summarizes the types of tumors from the cohorts, 2/20 for AA and 0/18 for CA (data not available for 2 cases). Together, these findings suggest that the high expression of *CD274* and *CTLA4* in the CA cohort are not due to up-regulation of their co-stimulatory molecules or due to a higher number of MSI tumors when compared to the AA group.

### Systemic Cytokine Secretion in African American and Caucasian American Colon Cancer Patients Matches Gene Expression Data

Given the differences in gene expression patterns between the two ethnic groups in this study, we examined if circulating levels of the encoded cytokines also differed between AA and CA colon cancer patients. ELISA assays (Multi-Analyte plates, 12 cytokines included) were used to detect cytokines involved in the activation, recruitment, and differentiation of T cells that could influence the cellular anti-tumor activity at the tumor site. As shown in Figure 4C, AA and CA colon cancer patients secreted significantly different types and levels of cytokines. The data collected from these assays was discriminated based on cytokines with values over the positive control and differences of significance (*p* < 0.05) between the groups (*n* = 40). For the correlation of the expression of cytokines to T cell subsets, we used the following markers; T_h_1 (IL-2, IL-12, INF-γ, TNF-α), T_h_2 (IL-4, IL-5, IL-10), T_h_17 (IL-17A, IL-6), and T Regulatory cells (T-Reg: IL-17A and TGF-β1).

CAs presented with a significant level of cytokines correlated to T_h_1 and T_h_2 activation, differentiation, and anti-tumor activity. However, it is imperative to mention that they did not present differential expression of the pro-inflammatory cytokines interleukin 17-A (IL-17A) and tumor necrosis factor alpha (TNF-α) when compared to AA patients. CA patients also presented with higher levels of the macrophage activator cytokine, G-CSF. Together, these results suggest that AA patients may fail to promote the anti-tumor activity of the T_h_1 and T_h_2 subsets of T cells and, similar to CA tumors, secrete cytokines associated with the presence of T_h_17 and inflammation.

### Comparison of the Gene Expression Levels From African American and Caucasian American Patients With the TCGA Database

**Figure 5:**
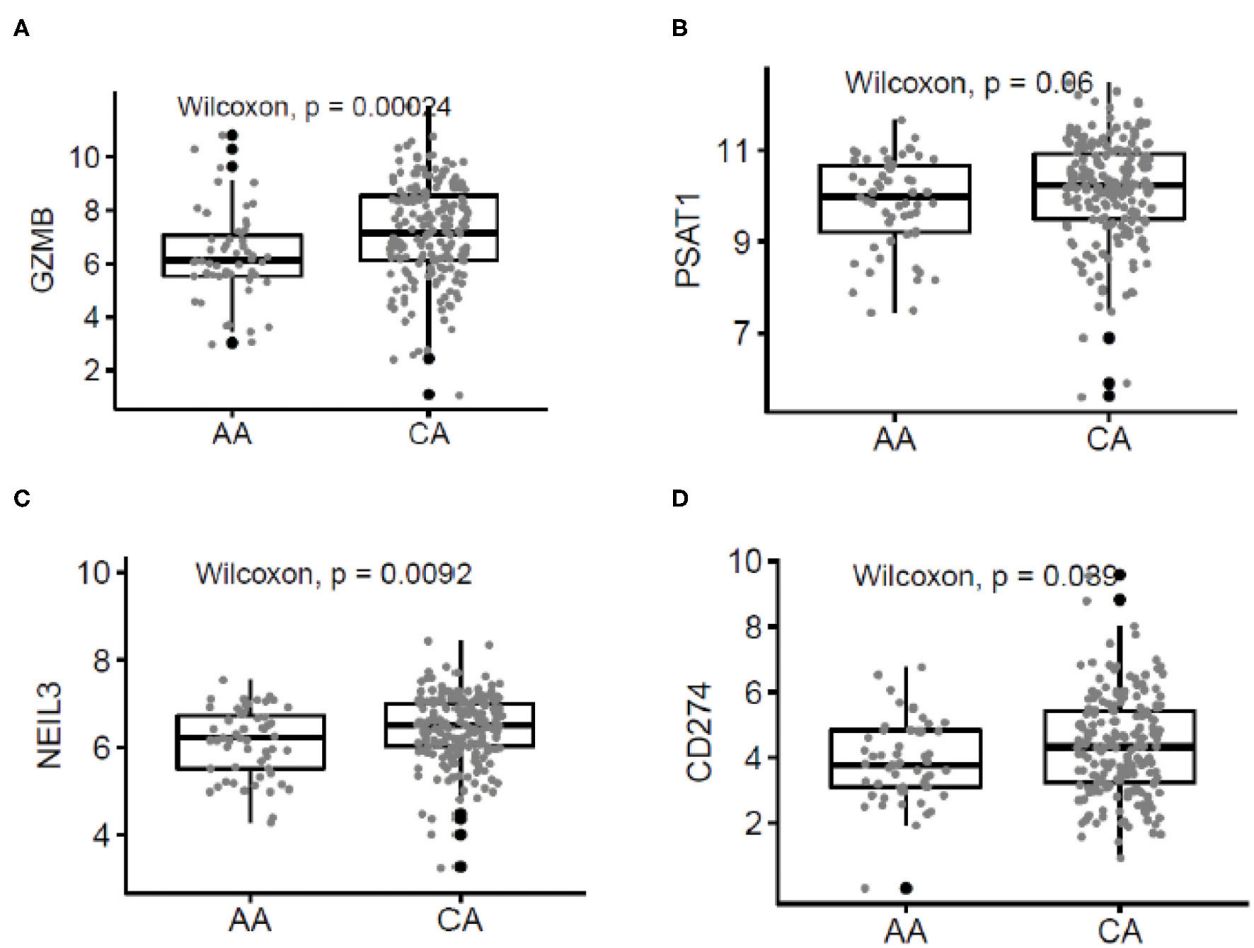
Correlated patterns of gene expression between the AA and CA cohorts and The Cancer Genome Atlas (TCGA). **(A–D)** Our gene expression findings correlate with the gene expression patterns from AA (*n* = 55) and CA (*n* = 193) colon cancer patients from the TCGA. *GZMB, PSAT1, NEIL3*, and *CD274* were upregulated in CAs when compared with AAs.

To validate our findings, we used the publicly available data on colon cancer from The Cancer Genome Atlas (TCGA) database. Figure 5 describes the correlation of selected genes up-regulated in tumors from CA when compared to AA such as higher expression of *GZMB* (*p* = 0.00024), *PSTAT* (*p* = 0.06), *NEIL3* (*p* = 0.0092), and *CD274* (*p* = 0.039). Importantly, the most significant difference in gene expression between the two cohorts in the TCGA data was *GZMB*. Therefore, we investigated if the expression of *GZMB* was associated with a difference in survival rates. Supplementary Figures 2D,E show, even though not significant, AA with higher levels of expression of *GZMB*, have better 5 year survival rates, results that differ from the survival rates in CAs. These results suggest that high expression of *GZMB* appears to be beneficial for AA patients exclusively and highlights the importance of analyzing each racial group separately and of increasing the number of samples from AA patients available in publicly accessible databases.

### Colon Tumors From African American Patients Showed an Impaired Infiltration of Cytotoxic Cells When Compared to Colon Tumors From Caucasian Americans

To further explain the differential immune gene expression observed between AA and CA with CRC we ran the NanoString CancerImmune Panel to predict the immune cell infiltration in those tumor samples.

**Figure 6:**
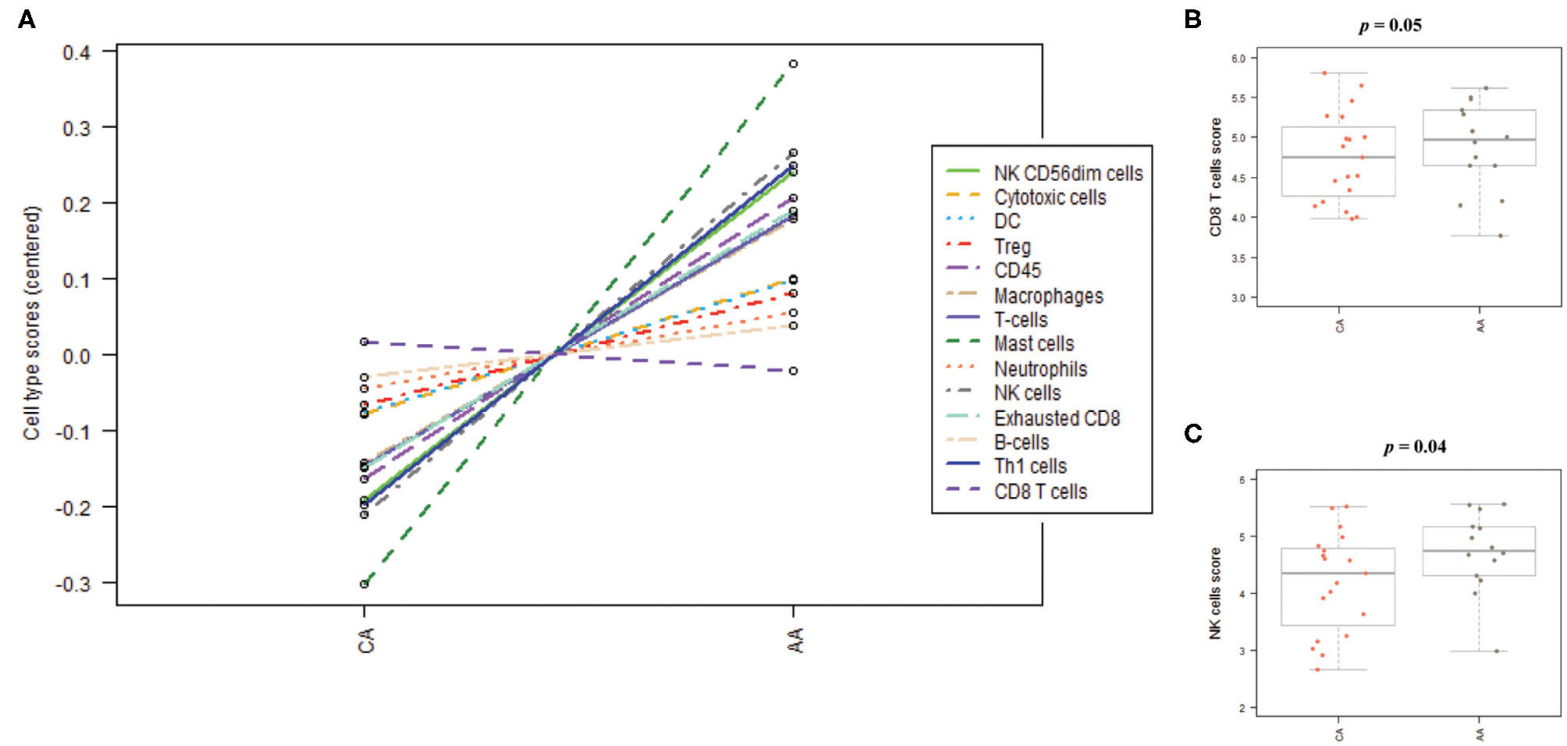
Immune cells distribution in colon tumors from African American and Caucasian American patients based on gene expression scores. **(A)** Cells' populations by cell type in each cohort. **(B)** Colon tumors from AA and CA patients showed not significant differences in the scores of CD8 positive T cells. **(C)** AA tumors presented significantly higher score of Natural Killer (NK) cells when compared to CA. Estimation of cells' infiltration was done with the nSolver software v4.0 (NanoString) based on the expression of cell specific transcripts normalized to several housekeeping genes. *p* < 0.05.

**Figure 7:**
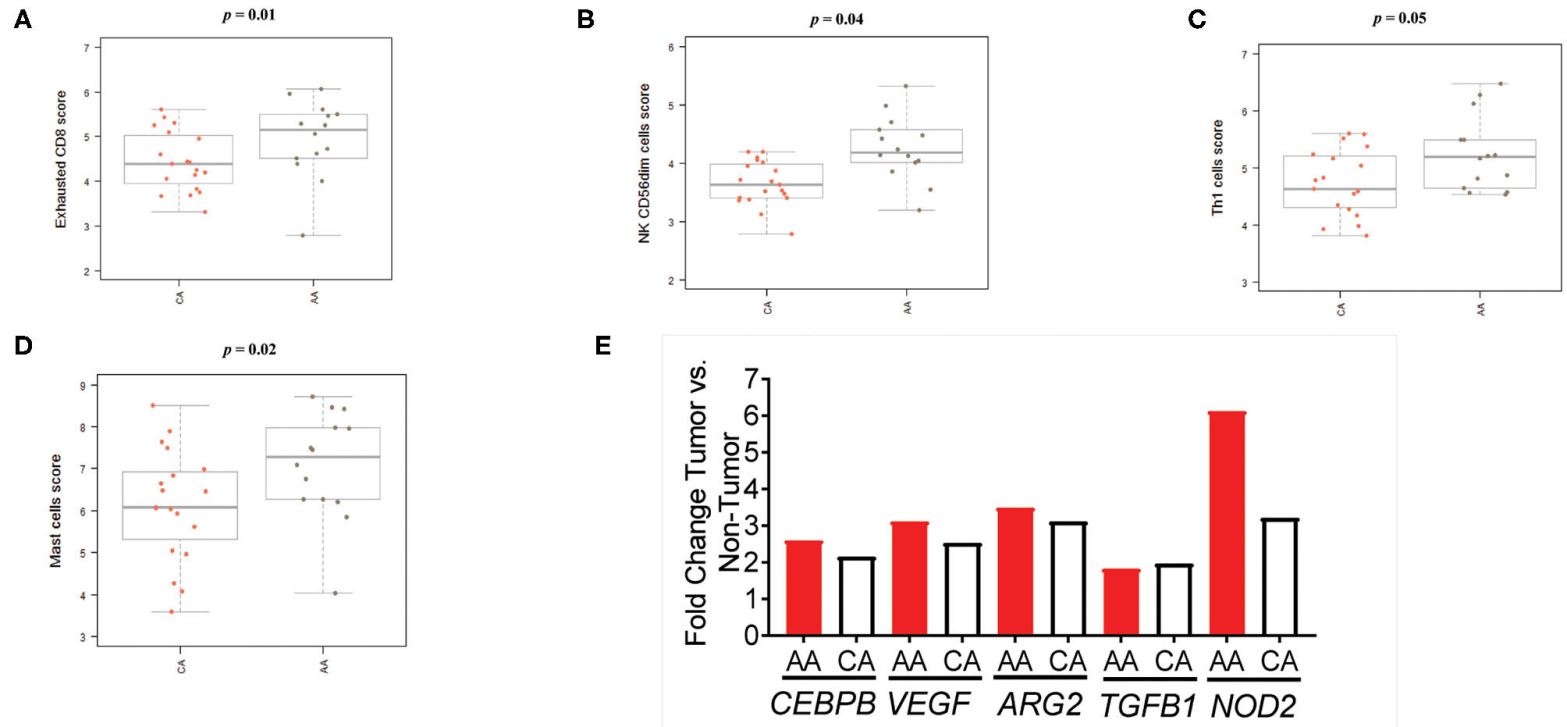
Colon tumors from AA patients present an impaired immunological response in terms of immune cell activation. **(A)** CD8+ T cells with markers related to cell exhaustion (increased expression of CD8, CD244, EOMES, LAG3) are significantly higher in the AA cohort. **(B)** The score of the cytotoxic NK sub-set, CD56dim, was significantly higher in tumors from AA patients. **(C)** The score of the T cells' sub-set Th1 in the AA cohort significantly higher than the score in CA. **(D)** AA tumors appear to have a significantly higher score of mast cells than tumors from CAs. **(E)** Tumors from AAs present upregulation of several genes associated with the presence of myeloid suppressor cells. Estimation of cells' infiltration was done with the nSolver software v4.0 (NanoString) based on the expression of cell specific transcripts normalized to several housekeeping genes. *p* < 0.05.

As depicted in Figure 6A, the scores of the majority of immune cell populations varied between the two groups. Remarkably, AA and CA tumors displayed similar scores of CD45 cells, a marker for leukocytes (*p* = 0.9). When we examined the cell sub-sets with potential cytotoxic activity, we found that AA tumors had significantly higher scores of CD8+ T cells (*p* = 0.05) and natural killer (NK) cells (*p* = 0.04) (Figures 6B,C, respectively). African American colon tumors also had a significant increase scores of exhausted CD8+ cells (*p* = 0.01), NK CD56dim cells (*p* = 0.04), and T_h1_ cells (*p* = 0.05) (Figures 7A–C, respectively). The latter set of data suggests that tumors from AAs present significant numbers of cytotoxic NK cells and their reduced cytotoxic activity is specific for CD8+ T cells.

We then proceeded to calculate the scores for other populations of immune cells, including myeloid cells, known to contribute to T-cell suppression (18–20). We found a significant increase in the score of mast cells (*p* = 0.02) in AA tumors when compared to CA (Figure 7D). These cells are associated with colon tumor growth and chronic inflammation (18–20). In addition, although there were not significant differences among the scores of dendritic cells, macrophages, neutrophils or B cells, they appeared to be upregulated in AAs when compared to the CA group (Supplementary Figure 4). Consequently, since immune-suppressive activity by promotion of inflammatory processes at the tumor site is one of the main characteristics of myeloid-derived suppressive cells (MDSCs) (18–20), we investigated how the gene expression in colon tumors relate to makers of MDSCs activation. Figure 7E demonstrates that five genes coding for proteins associated with MDSCs and inflammation are significantly expressed in tumors from both groups but AAs exhibited a higher fold-change in all genes with the highest difference being for *NOD2* expression (2.0-fold over CA). This led us to propose that tumors from AAs have an increased expression of MDSC-related genes as compared to tumors from CAs and that the presence of myeloid cells may contribute to the secretion of pro-inflammatory cytokines and chronic inflammation at the tumor site.

Taking our gene expression and NanoString data together, we infer that AA colon cancer patients present a reduced cytotoxic, antitumoral activity at the tumor site when compared to CAs.

## Discussion

In this study, we discovered potential immune markers for colon cancer prognosis and targets for therapies that could lessen the persistent disparities observed between AA and CA colon cancer patients. Our findings demonstrate that colon tumors from AA and CA patients differ in the number, type, and level of expression of several immunological genes and immune cells associated with colon cancer. Specifically, our results indicate that AA colon tumors gene expression is associated with an impaired immune response and diminished antitumor activity when compared to the tumors from CA colon cancer patients. This impairment was suggested by the downregulation of several markers for cytotoxic T cells as well as upregulation of multiple markers associated with inflammation and the presence of exhausted CD8+ and T regulatory cells. Although past studies have reported differences in either the germline or somatic alterations in genes of tumors from AAs as compared to tumors from CAs, including alterations in the *KRAS, BRAF*, and *PIK3CA* genes (3–6), we believe our study is the first to collectively investigate the role of the immunological differences between these populations in terms of gene expression, immune cell recruitment and cytokines levels in plasma. The results presented here suggest a reduced antitumor activity in AAs when compared with CA colon cancer patients.

One of the top targets found to be significantly different in AA and CA samples was *GZMB* which encodes a member of the Granzyme subfamily of proteins, part of the peptidase S1 family of serine proteases (15). The encoded preproprotein is secreted by natural killer (NK) cells and cytotoxic T lymphocytes and is processed to generate the active protease, which induces target cell apoptosis (15). This protein also processes cytokines and degrades extracellular matrix proteins, roles that have been implicated in chronic inflammation, wound healing, and tumor regression (15). Furthermore, we found consistency in the results from CA colon tumors that have been sequenced and made available in the TCGA database. Not surprisingly, Granzyme B has been proposed as a biomarker to predict patient response to immunotherapies where an upregulation of *GZMB* would be associated with increased cytotoxic activity and tumor size reduction (15). As such, we are proposing this gene/protein as a potential therapeutic target for African American colon cancer patients.

In line with the downregulation of *GZMB* in colon tumors from AAs, we also observed an upregulation of exhausted CD8+, T regulatory and myeloid cells in this cohort when compared to CAs. The number and type of immune cell populations that infiltrate the tumor site and how they relate to colon cancer has been studied previously (21–23). For instance, the presence of T regulatory cells in tumors was correlated to immune suppression, poor outcome, and increased tumor size for colon cancer (21–23). Cytotoxic T cells in turn, such as CD8+ T cells, have been associated with higher survival rates as well as enhanced responses to immunotherapy treatment and lower recurrence rates (13, 14, 22). Accordingly, low infiltration of cytotoxic T cells (CD8^+^) or infiltration of these cells with markers of cell exhaustion, result in low levels of antitumor immunity (21). Hence, we wanted to address if African American patients diverge in the number and activation markers of immunological cells when compared to their Caucasian American counterparts. By investigating if colon tumors from African American patients (MSI and MSS included) showed a different pattern of activation of CD8+ T cells, recruitment of myeloid and other immune cells, we aimed to elucidate the disparities between the cellular immune response among African American and Caucasian American colon cancer patients.

Remarkably, we found that colon tumors from African American patients had a significantly higher cell score of Exhausted (impaired) CD8+ T cells (*p* = 0.01) than tumors from Caucasian Americans. Although they presented a significantly higher score of overall T_h_1 T cells (*p* = 0.05) and cytotoxic NK CD56_dim_ cells (*p* = 0.04), the presence of exhausted CD8+ T cells suggest that T cells are recruited to the tumor site but lack activation as indicated by the gene expression of biomarkers indicative of T cell exhaustion such as *CD8, CD244, EOMES*, and *LAG3*.

Further possible explanations for the significantly high score of exhausted CD8 + T cells in the AA cohort when compared to tumors from CA patients, include the capacity of T regulatory cells to exhaust CD8+ cells by depletion of cytokines such as IL-10 and IL-12 (23). We found that AA tumors had a higher score for T regulatory cells and a significantly higher gene expression level of *FOXP3*, marker of regulatory T cells (validated by qPCR) when compared to the CA group. In agreement with these results, we also demonstrated that AA and CA patients had a significant difference (*p* < 0.05) in plasma cytokine T_h_1/T_h_2/T_h_17 concentrations by ELISA. Correlating with the upregulation of markers associated with cytotoxic T cells in CA tumors, such as the gene expression of *GZMB* and *IFNG*, we saw significantly higher levels of the cytokines IL-12 and IL-10 in plasma from the CA patients. IL-10 is involved in the maintenance of immune homeostasis in the colon and the activation of CD8^+^ cytotoxic T-lymphocytes (16). Similarly, IL-12 is capable of enhancing IFN-γ-producing CD8^+^ T-cell numbers at the tumor site and consequently, to suppress tumor growth (16). Importantly, administration (subcutaneous injections) of these cytokines in combination were proven to increase the number and activity of cytotoxic CD8^+^ cells, to decrease tumor growth and to enhance survival in mice, suggesting that these cytokines can act in concert to improve outcomes in colon cancer (16). Collectively, our cytokines secretion, cell recruitment and gene expression findings suggest that CA patients may present a more effective CD8^+^ antitumor activity when compared to the AA cohort.

We also investigated myeloid cells and genes linked to their immunosuppressive activity in the AA cohort that could explain the reduced number of functional cytotoxic T cells in the tumors from AAs. Myeloid cells are a highly diverse population that includes macrophages, dendritic cells, neutrophils and mast cells among others (24). These cells are capable to respond to and secrete IL-1β, IL-8 among other pro-inflammatory cytokines, and are associated with inhibition of T cell activation (24–26). As part of their immunosuppressive mechanisms are the downregulation of IFN-γ, and the production of molecules that contribute to chronic inflammation (11, 16, 17). For example, our results demonstrate that AA tumors presented a significantly higher score of mast cells (*p* = 0.02), which have been previously associated with immunosuppression in colon cancer and worse outcome (24). In conjunction, we examined the expression of genes coding for proteins associated with myeloid-derived suppressor cells (MDSCs) that promote pro-inflammatory cytokine secretion, bowel inflammation and nitric oxide production in the colon (16, 17). In agreement with the observed myeloid cells scores, we observed that the levels of *CEBPB* (CCAAT/enhancer-binding protein beta), *VEGF* (Vascular endothelial growth factor), *TGFB1* (Transforming growth factor beta 1) and *NOD2* (Nucleotide-binding oligomerization domain-containing protein 2) were significantly higher in tumors from AAs (as compared to their non-tumor counterpart). Hence, by associating the scores of myeloid cells and the level of expression of genes coding for molecules produced by MDSCs, we suggest that the presence of myeloid cells could contribute to the impairment of antitumor activity in tumors from AA patients.

Taking into consideration our results indicating that colon tumors from AA patients appear to have an impaired cytotoxic tumor environment, we examined the expression of immunotherapy targets Programmed cell Death-1 (PD-L1) and cytotoxic T-lymphocyte-associated protein 4 (CTLA-4) in our samples. Hence, it is paramount to highlight that our gene expression results and the ones obtained from The Cancer Genome Atlas Program (27) showed significantly lower levels of gene expression of *PDL1* (*CD274*) in the AA colon tumors when compared to the CA cohort. Notably, our results also revealed the significantly lower expression of *CTLA4*, the immunotherapy blockade target that prevents T regulatory cells immunosuppression (28) in the AA tumors. In addition, as microsatellite instability (MSI) is one biomarker for response to PD-L1 and CTLA-4 immunotherapies and good prognosis in colon cancer (28–31), we explored the frequency of MSI to predict the number of AA patients that could benefit from these immunotherapies that appear to be effective in CA colon cancer patients (28–31). Our molecular tumor classification discovered that although 10% of the tumors from AA were MSI and CA tumors were 100% MSS (2 CA samples were unknown), CA patients may benefit more than AAs from the immunotherapies available to date.

In this study, we demonstrated by whole transcriptome analysis that colon tumors from AAs present a reduced expression of several genes associated with cytotoxic and antitumor activity and an upregulation of pro-inflammatory and immunosuppressive genes. We also propose that colon tumors from AA patients have a significantly higher population of impaired cytotoxic T cells as well as mast and T regulatory cells. In addition, we provided data on the significantly different patterns of systemic secretion of cytokines that could contribute to the immunological disparities observed between these two populations. Lastly, we showed a correlation between our gene expression findings and the ones available from TCGA that demonstrated that Granzyme B could be a therapeutic target for AA colon cancer patients. Although our limitations included a small sample size and missing other molecular analysis, such as DNA sequencing that may elucidate the gene expression differences here presented; we believe these initial findings can serve as a model for future investigations on racial cancer disparities.

In conclusion, the results presented highlight the need to investigate the potential use of alternative immunotherapies for colon cancer that could benefit AA patients regardless of their MSI status and expression of the *PDL1* and *CTLA4* genes. For instance, we propose to further investigate the role of MDSCs and their metabolic products in the immunosuppressive tumor microenvironment in AA colon cancer patients. Also, mutational analysis of the studied colon tumors may help us to elucidate the gene expression differences that we herein report. Thus, since we observed an up to 1 to 10 ratio of differential gene expression between CA and AA colon tumors for the *IL1B* and *IL8* genes, our future studies will explore the relationship between the cytokine secretion patterns in AA tumors as well as in colon cancer cell lines from AA patients (32).

## Data Availability Statement

The raw data (FASTQ) generated for this study can be found in the Gene Expression Omnibus (GEO) with the accession number GSE146009.

## Ethics Statement

The studies involving human participants were reviewed and approved by SUNY Downstate Medical Center and Kings County Hospital. IRB Protocol #312509. The patients/participants provided their written informed consent to participate in this study.

## Author Contributions

JP, JZ, LM, JW, and JC: contributed conception and design of the study, critical revision of the manuscript, and study supervision. JP, PJ, SI, MAk, KB, MM-S, RG, MAl, MAg, and HT: obtaining and processing of samples. JP, JZ, XW, LM, and JW: analysis and interpretation of data. JP, JZ, JG, JA, LL, KB, XW, and MS: data acquisition. JP, JZ, LM, JW, and JC: wrote sections of the manuscript. All authors contributed to manuscript revision, read, and approved the submitted version.

## Conflict of Interest

The authors declare that the research was conducted in the absence of any commercial or financial relationships that could be construed as a potential conflict of interest.

## Supplementary Material

Supplementary Figure 1 AA and CA tumors commonly expressed the Cytokine-Cytokine Receptor Interaction pathway but differ in the expression pattern. For example, CA tumors **(B)** have an increased number of downregulated CC chemokine genes (green boxes) upregulated CXC chemokine genes (red boxes) when compared to AA tumors **(A)**. Oher differences can be seen in genes belonging to the TNF and TGFβ families Pathways were generated with the KEGG algorithm in Partek Flow. Red boxes indicate upregulation of genes in the tumor tissues as compared to the adjacent non-tumor tissues. Green boxes indicate downregulation of the gene in the tumor.

Supplementary Figure 2 Validation of the RNA sequencing data by Real Time PCR. **(A–C)** Gene expression results (whole transcriptome) of the *GZMB, FOXP3*, and *IL1B* genes were validated by Real time PCR. RNA was isolated from AA and CA colon tumors and their corresponding non-tumor controls. *P* < 0.05. *GAPDH* was used as housekeeping gene and the relative expression was determined based on the expression of the corresponding non-tumor tissues by the 2^−ΔΔCT^ method. **(D)** High expression of *GZMB* (Granzyme B) in tumors correlates with better overall survival in AAs (*n* = 55) *P* = >0.05. Kaplan-Meier survival curve based on expression of GZMB in using data extracted from the TCGA. **(E)** High expression of GZMB in tumors from CA (*n* = 193) showed no correlation with better overall survival for colon cancer. Kaplan-Meier survival curve based on expression of *GZMB* using data extracted from the TCGA (Up-regulated vs. others).

Supplementary Figure 3 CA tumors significantly expressed the DNA repair pathway. The CA cohort presented a significant up-regulation of genes associated with DNA repair, including genes from the mismatch repair family, MMR. AA tumors did not significantly express this pathway over their non-tumor tissue (control). Pathways were generated as explained for Supplementary Figure 2.

Supplementary Figure 4 AA and CA differ in the cellular score of several immune cells' sub-types recruited to the tumor site. **(A)** The expression of CD45 (lymphocyte's marker) does not significantly differ between the two cohorts. **(B)** Although not significant, AA appear to have a higher number of T regulatory cells at the tumor site. **(C)** The cytolytic cellular scores (includes T and NK cells) were not significantly different between the two groups. **(D–G)** The score of dendritic cells (DCs), macrophages, neutrophils, and B cells were not significantly different between the two groups. Estimation of cells' infiltration was done with the nSolver software v4.0 (NanoString) based on the expression of cell specific transcripts normalized to several housekeeping genes. *p* < 0.05.

Supplementary Table 1 Patients characteristics^*^.
