# Supplementary figures and images for: Immune-Related Gene Expression and Cytokine Secretion Is Reduced Among African American Colon Cancer Patients
Source: Front Oncol. 2020 Sep 2;10:1498. doi: 10.3389/fonc.2020.01498 (PMC7492388; doi:10.3389/fonc.2020.01498)

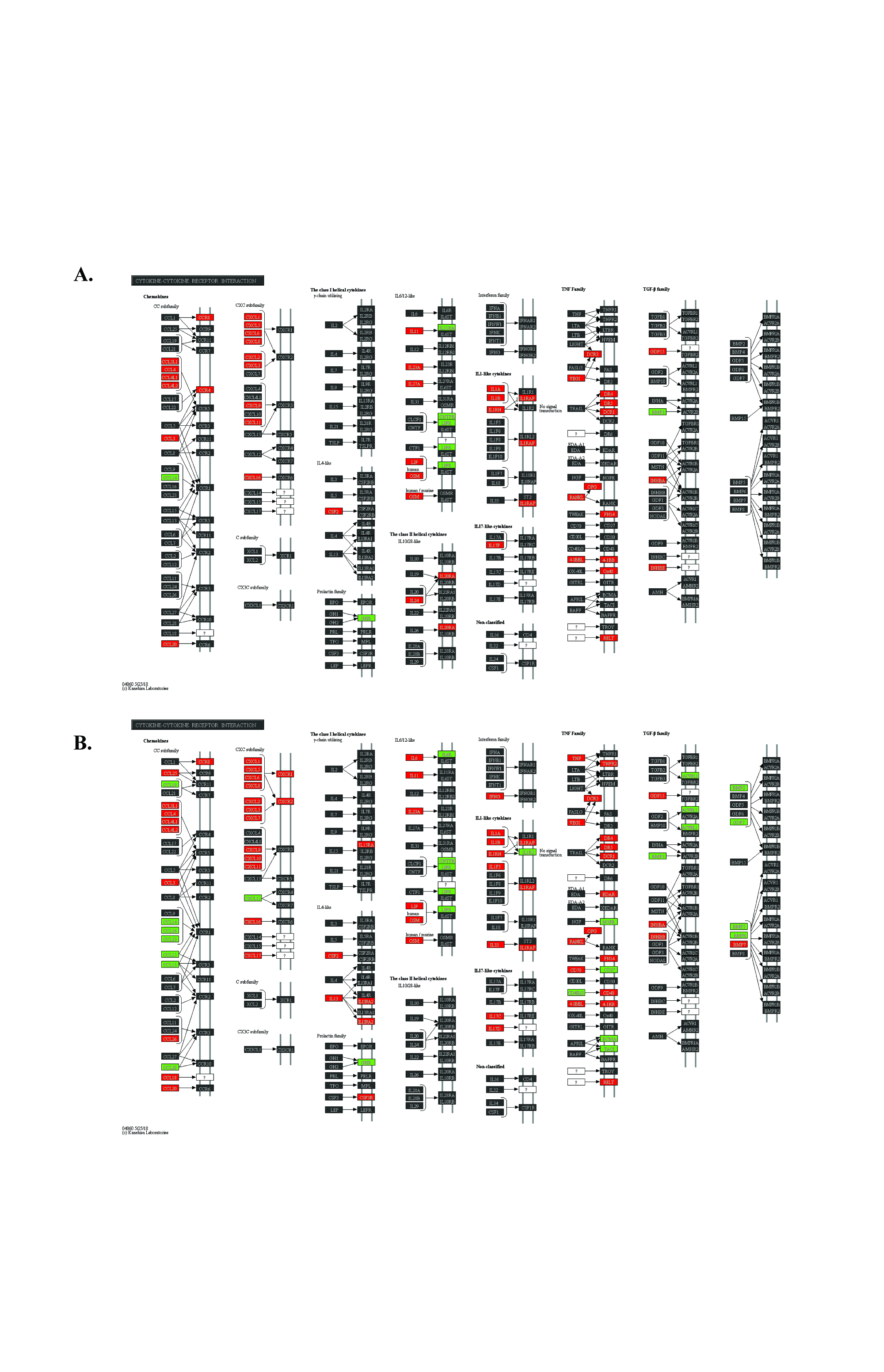

Supplement: Supplementary Figure 1 — AA and CA tumors commonly expressed the Cytokine-Cytokine Receptor Interaction pathway but differ in the expression pattern. For example, CA tumors (B) have an increased number of downregulated CC chemokine genes (green boxes) upregulated CXC chemokine genes (red boxes) when compared to AA tumors (A). Oher differences can be seen in genes belonging to the TNF and TGFβ families Pathways were generated with the KEGG algorithm in Partek Flow. Red boxes indicate upregulation of genes in the tumor tissues as compared to the adjacent non-tumor tissues. Green boxes indicate downregulation of the gene in the tumor. [file Image_1.TIF]

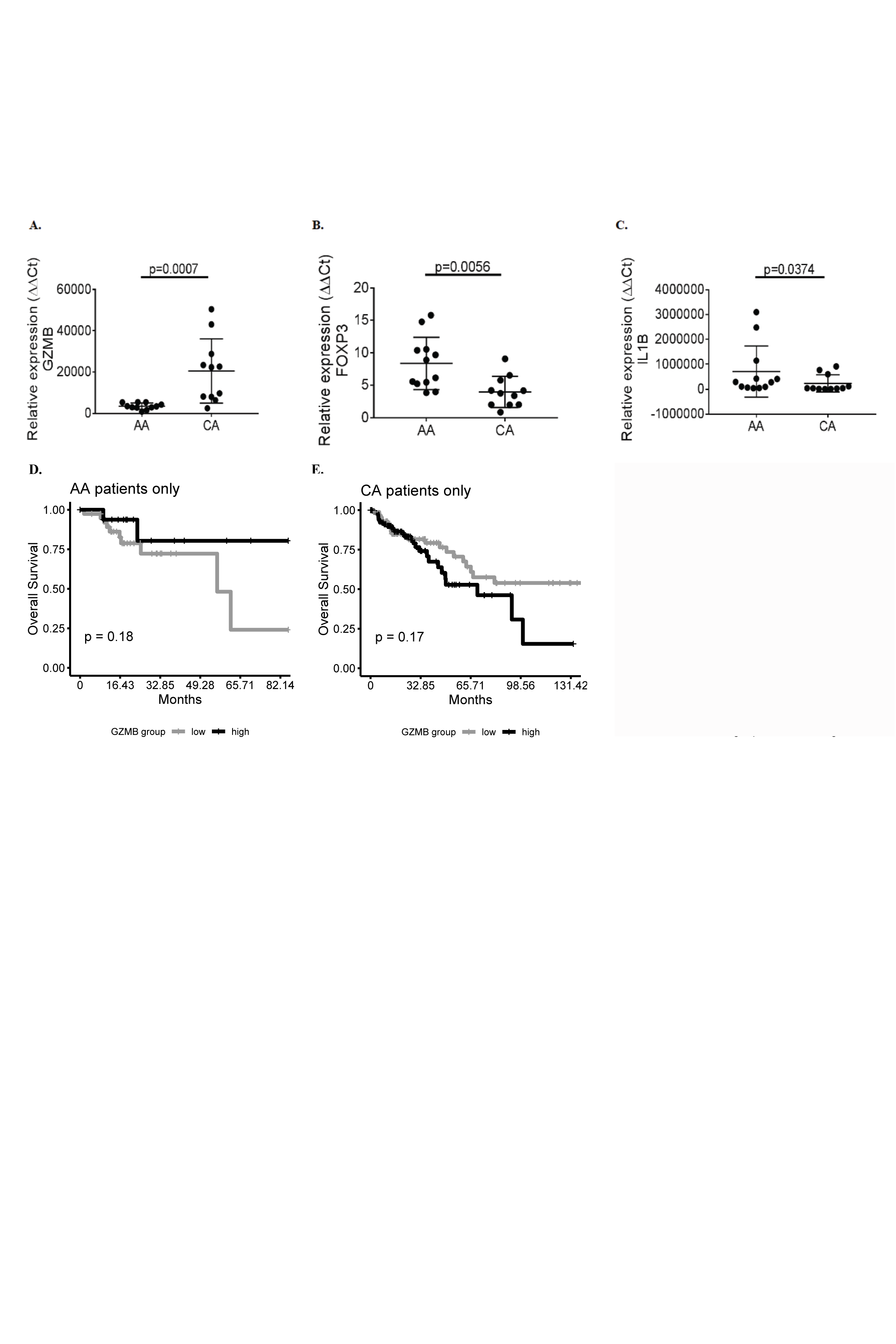

Supplement: Supplementary Figure 2 — Validation of the RNA sequencing data by Real Time PCR. (A–C) Gene expression results (whole transcriptome) of the GZMB, FOXP3, and IL1B genes were validated by Real time PCR. RNA was isolated from AA and CA colon tumors and their corresponding non-tumor controls. P < 0.05. GAPDH was used as housekeeping gene and the relative expression was determined based on the expression of the corresponding non-tumor tissues by the 2−ΔΔCT method. (D) High expression of GZMB (Granzyme B) in tumors correlates with better overall survival in AAs (n = 55) P = >0.05. Kaplan-Meier survival curve based on expression of GZMB in using data extracted from the TCGA. (E) High expression of GZMB in tumors from CA (n = 193) showed no correlation with better overall survival for colon cancer. Kaplan-Meier survival curve based on expression of GZMB using data extracted from the TCGA (Up-regulated vs. others). [file Image_2.TIF]

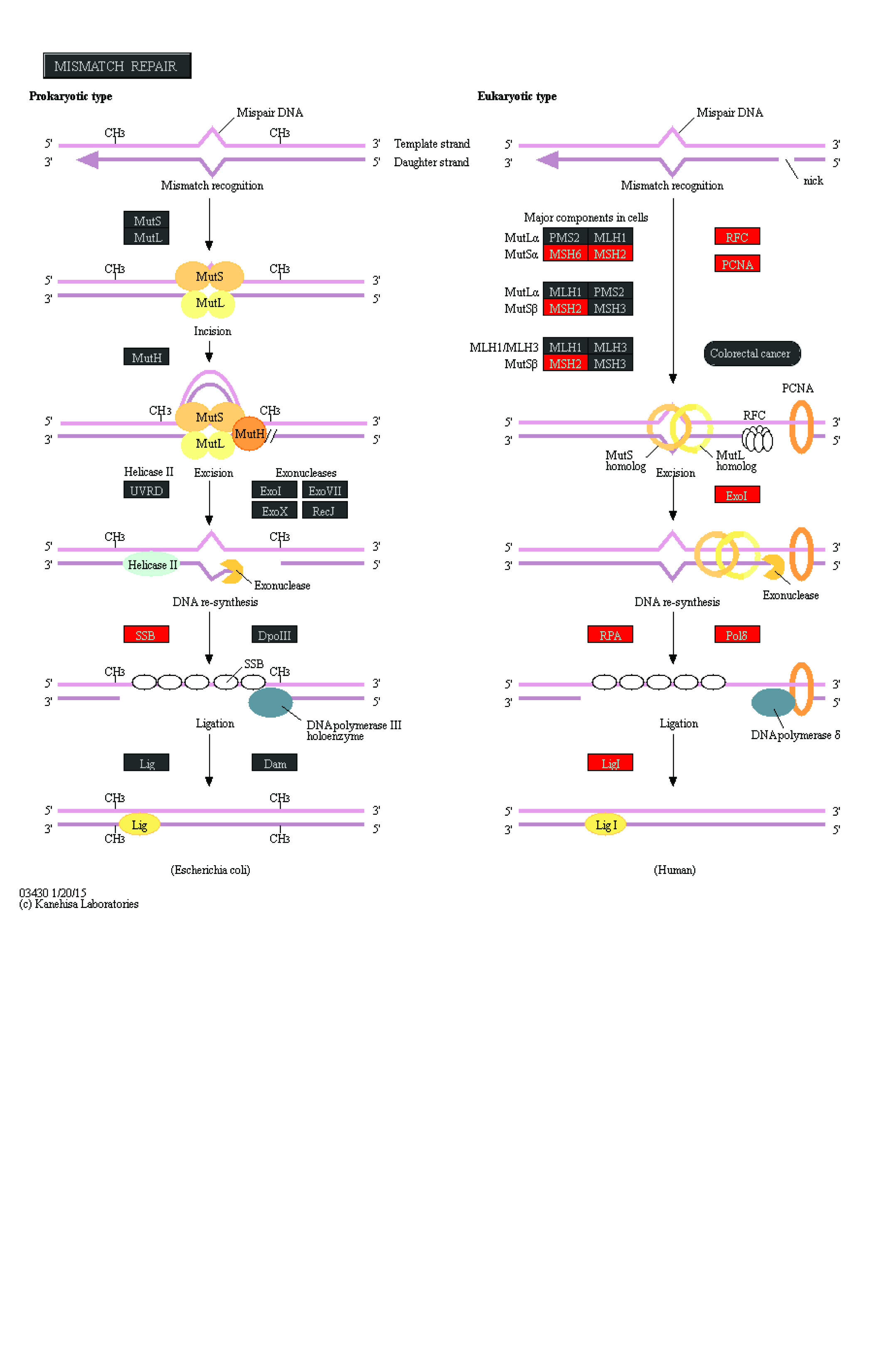

Supplement: Supplementary Figure 3 — CA tumors significantly expressed the DNA repair pathway. The CA cohort presented a significant up-regulation of genes associated with DNA repair, including genes from the mismatch repair family, MMR. AA tumors did not significantly express this pathway over their non-tumor tissue (control). Pathways were generated as explained for Supplementary Figure 2. [file Image_3.TIF]

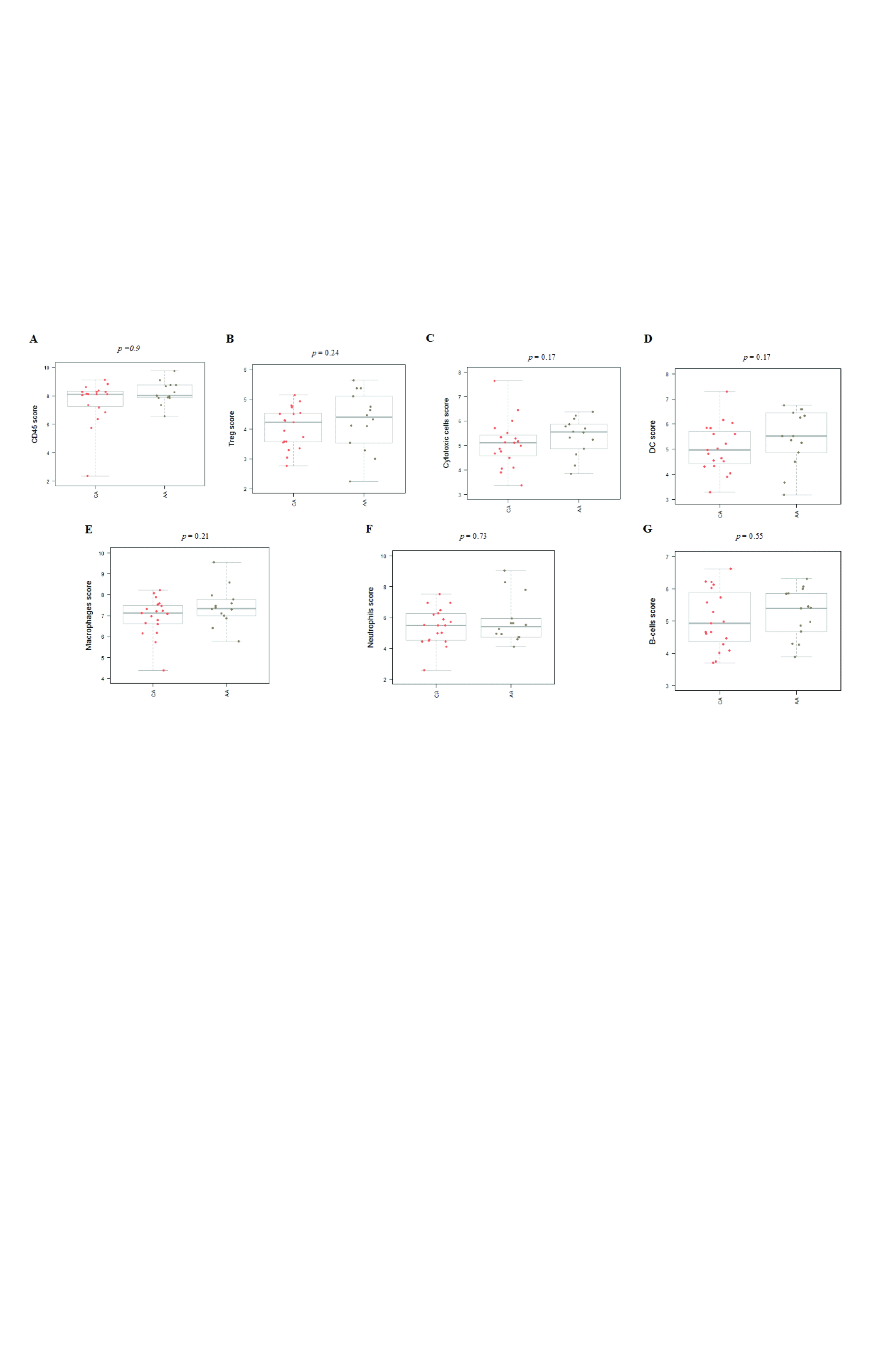

Supplement: Supplementary Figure 4 — AA and CA differ in the cellular score of several immune cells' sub-types recruited to the tumor site. (A) The expression of CD45 (lymphocyte's marker) does not significantly differ between the two cohorts. (B) Although not significant, AA appear to have a higher number of T regulatory cells at the tumor site. (C) The cytolytic cellular scores (includes T and NK cells) were not significantly different between the two groups. (D–G) The score of dendritic cells (DCs), macrophages, neutrophils, and B cells were not significantly different between the two groups. Estimation of cells' infiltration was done with the nSolver software v4.0 (NanoString) based on the expression of cell specific transcripts normalized to several housekeeping genes. p < 0.05. [file Image_4.TIF]
