# Supplementary material for: Immune-Related Gene Expression and Cytokine Secretion Is Reduced Among African American Colon Cancer Patients
Source: Front Oncol. 2020 Sep 2;10:1498. doi: 10.3389/fonc.2020.01498 (PMC7492388; doi:10.3389/fonc.2020.01498)
Supplement: Supplementary Table 1 — Patients characteristics*. [file Table_1.docx]

**Supplementary Table 1**. Patients Characteristics*

| **Race** | **Sex** | **Age Range** | **Age Average** | **Age *P*** **Value**  **AA vs. CA** | **Tumor Stage**** |
| --- | --- | --- | --- | --- | --- |
| **African**  **Americans** | Female  7 | 46-76 | 62.4 | 0.86  **NS** | Stage I  3 |
|  | Male  13 |  |  |  | Stage II  6 |
| N of AA participants= 20  (RNAseq for 15/20; 20/20 for ELISA) | | | |  | Stage III  11 |
| **Caucasian Americans** | Female  8 | 45-90 | 64.7 |  | Stage I  5 |
|  | Male  12 |  |  |  | Stage II  5 |
| N of CA participants= 20  (RNAseq for 18/20; 20/20 for ELISA) | | | |  | Stage III  10 |

*Information from colon cancer patients that self-identified as African American or Caucasian American.

**Tumors Stage IV were excluded from the study due to the use of adjuvant therapy.
